# The migration of physicians from sub-Saharan Africa to the United States of America: measures of the African brain drain

**DOI:** 10.1186/1478-4491-2-17

**Published:** 2004-12-14

**Authors:** Amy Hagopian, Matthew J Thompson, Meredith Fordyce, Karin E Johnson, L Gary Hart

**Affiliations:** 1 WWAMI Center for Health Workforce Studies, Department of Family Medicine, University of Washington, Seattle, Washington, USA; 2 Department of Primary Health Care, University of Oxford, Oxford, UK

## Abstract

**Background:**

The objective of this paper is to describe the numbers, characteristics, and trends in the migration to the United States of physicians trained in sub-Saharan Africa.

**Methods:**

We used the American Medical Association 2002 Masterfile to identify and describe physicians who received their medical training in sub-Saharan Africa and are currently practicing in the USA.

**Results:**

More than 23% of America's 771 491 physicians received their medical training outside the USA, the majority (64%) in low-income or lower middle-income countries. A total of 5334 physicians from sub-Saharan Africa are in that group, a number that represents more than 6% of the physicians practicing in sub-Saharan Africa now. Nearly 86% of these Africans practicing in the USA originate from only three countries: Nigeria, South Africa and Ghana. Furthermore, 79% were trained at only 10 medical schools.

**Conclusions:**

Physician migration from poor countries to rich ones contributes to worldwide health workforce imbalances that may be detrimental to the health systems of source countries. The migration of over 5000 doctors from sub-Saharan Africa to the USA has had a significantly negative effect on the doctor-to-population ratio of Africa. The finding that the bulk of migration occurs from only a few countries and medical schools suggests policy interventions in only a few locations could be effective in stemming the brain drain.

## Background

Doctors migrate from developing countries to wealthier countries in order to further their careers, or improve their economic or social situation. The World Health Organization (WHO) has long recognized that migration of health personnel from developing to developed countries creates unfortunate imbalances in the global health workforce [1].

America's physician workforce has been significantly infused with foreign-trained international medical graduates (IMGs) since World War II. The purpose of this paper is to describe a sub-population of IMGs in the USA, those who have trained in one of the 47 African subcontinent nations.

African governments have been very clear about their objections to the wholesale migration of their physicians to rich countries. In 1996, South Africa's then-Deputy President Thabo Mbeki implored the World Health Assembly to take measures to stop the flow of physicians from poor countries to rich ones. In 1995, South Africa itself banned the recruitment of doctors from other Organization of African Unity countries [2].

Nonetheless, large numbers of African-trained physicians leave home upon completion of their medical school training in search of careers in higher-income countries. They leave behind health systems in sub-Saharan Africa that are severely stressed: life expectancy is only 50 years, 162 children in 1000 die before reaching their fifth birthdays, and only half have access to clean water sources [3]. Further, AIDS prevalence among those 15 to 49 years old is estimated to be 8.4% [4], and in four countries, adult HIV prevalence exceeds 30% [5]. While health improvements in Africa will require a broad agenda of development activities, access to an educated workforce of health professionals is also essential [6].

African country health systems and workforce data are poor, making it difficult to estimate the effects of physician migration on sending countries. The World Bank has documented this data gap, noting "Quantitative data on the health workforce is notoriously unreliable in most countries...In poor countries, government and professional information systems are weak, when they exist at all, and are rarely comprehensive (often there is no information on the private sector) and up-to-date" [7]. Indeed, the way many African country ministries of health learn about the extent of their own emigration is through gleaning data presented by destination countries [8]. This paucity of sending-country data makes it difficult to fully describe the impact of migration on countries of origin.

The 47 nations of sub-Saharan Africa have a total of 87 medical schools, although 11 countries have no medical school at all and 24 have only one each (see Table 1). The population of the subcontinent totals over 660 million people, with a ratio of fewer than 13 physicians per 100 000 population, or a total of 82 949 doctors [9]. By comparison, the United Kingdom (UK) has 164 physicians per 100 000 and the USA has over 279 physicians per 100 000 (or almost 800 000 doctors for a population of 284 million).

**Table 1:** Physician workforce distribution and number of medical schools by African country

**Country**	**Population (in 1000s)**	**Physicians per 100 000 population**	**Total number of physicians**	**FAIMER number of medical schools**
Angola	10 132	7.7	780	1
Benin	6428	5.7	366	1
Botswana	1578	23.8	376	0
Burkina Faso	12 217	3.4	415	1
Burundi*	5714	6	343	1
Cameroon	14 792	7.4	1095	1
Cape Verde	0.04	17.1	68	0
Central African Republic	3501	3.5	123	1
Chad	8419	3.3	278	1
Comoros	0.578	7.4	43	0
Congo	2809	25.1	705	1
Congo (DR)	51 810	6.9	3575	3
Côte d'Ivoire	15 866	9	1428	1
Equatorial Guinea	0.474	24.6	117	0
Eritrea	4232	3	127	0
Ethiopia*	62 651	2	1253	3
Gabon*	1223	20	245	1
Ghana	19 509	6.2	1210	3
Guinea	8642	13	1123	1
Guinea-Bissau	1278	16.6	212	1
Kenya	30 310	13.2	4001	2
Lesotho	1847	5.4	100	0
Liberia	3149	2.3	72	2
Madagascar	15 506	10.7	1659	3
Malawi*	10 874	2.3	250	1
Mali	10 665	4.7	501	1
Mauritania	2668	13.8	368	0
Mauritius	1179	85	1002	1
Mozambique#	16 934	2.57	435	1
Namibia	11 826	29.5	3489	0
Niger	10 174	3.5	356	1
Nigeria	123 750	18.5	22 894	16
Rwanda*	7405	4	296	1
Sao Tome & Principe	0.16	46.7	75	0
Senegal	9784	7.5	734	2
Seychelles	0.08	132.4	106	1
Sierra Leone	5203	7.3	380	1
So. Africa	42 351	56.3	23 844	8
Somalia	7253	4	290	1
Sudan	35 080	9	3157	14
Swaziland	1,120	15.1	169	0
Tanzania	33 768	4.1	1384	4
The Gambia	1367	3.5	48	0
Togo	5033	7.6	383	1
Uganda*	23 496	3	705	3
Zambia	9799	6.9	676	1
Zimbabwe	12 186	13.9	1694	1
TOTAL/AVG	663 529	12.5	82,949	1.8 avg
Avg. physicians per country			1765	

*Indicates countries for which physicians/100 000 data came from World Bank instead of World Health Organization

# Mozambique data come from Stephen S. Gloyd, as no data are available from WHO or World Bank

Note on calculations: Average physicians per 100 000 population in Africa overall = total number of doctors (82 949)/total population (663 529)

Sources: Population data from United States Census Bureau IDB Summary international Data Base (United Nations and National Statistics Offices)

Number of physicians from the World Health Organization

Number of FAIMER (Foundation for the Advancement of International Medical Education and Research) medical schools comes from

The dependence of the United States on IMGs is encoded in various policies, most specifically Medicare's financial support for significantly more residency positions than we have domestic medical school graduates [10]. Additionally, the USA will waive the exchange visitor requirement that would otherwise return IMGs to their home countries after residency training in exchange for agreements to practice in underserved USA settings. Further, the USA will grant permanent residency status to IMGs under a variety of conditions [11].

The UK has initiated efforts to meet its own health workforce planning needs while paying attention to global equity considerations by adopting a formal "code of practice" that prohibits its National Health Service employers from recruiting health professionals from a long list of developing countries [12]. While this code has not resulted in a reduction in nurse recruitment, the number of physicians migrating to the UK has declined for a brief period (but is now back up) [8,13]. Recently, two prominent medical journals in the UK, the *Lancet *and the *British Medical Journal*, have editorialized on the effects of the brain drain in poor countries, recommending an international code of ethics prohibiting the recruitment of developing world health professionals by rich countries [14,15].

While the UK has a centralized health system well positioned to address these issues, both within its health care system and with representatives of other nations, the USA, in contrast, has a fractured health system that is less able to engage these issues. Agencies of the USA government have been reluctant, unable or unwilling to impede free-market driven physician migration.

United States policies have always been quite friendly to physician migration, even taking into account toughened medical licensing examinations and tightened immigration rules over the past four or five decades. Furthermore, even though some types of immigration have been more restricted since September 11, 2001, Congress subsequently expanded the number of foreign physicians who will be granted favorable immigration status (HR 2215, passed 10/3/02 increases the number of J-1 visa waivers allocated to state health departments from 20 to 30; further, the Department of Health and Human Services took over the role formerly played by the USA Department of Agriculture in handling applications of J-1 waivers, thereby ensuring additional foreign physicians will have access to waivers.).

One of the most common initial points of entry for IMG physicians into the USA medical workforce is residency training program enrollment, even if physicians have already completed postgraduate training in their home countries. The reliance of many inner-city hospitals on IMGs has thwarted calls by medical policy organizations, such as the Council on Graduate Medical Education, to reduce the number of IMGs admitted to residency programs as a means of narrowing the IMG pipeline to the USA

There is little debate within the USA government or other institutions about the social justice implications of obtaining health professionals from poor countries [16]. Typically, research on the issues surrounding the role(s) of IMGs in the USA has focused on 1)whether IMGs practicing here contribute to a surplus of physician labor (which could tend to lower physician salaries and/or drive up health care costs) [17-19]; 2) the quality of care delivered by IMGs [20]; and 3) the contribution of IMGs to the "health safety net" in rural or underserved areas [21].

The ethics of health professional migration from poor countries to rich ones is complicated by the competition of legitimate interests – each country's need for an adequate health workforce as opposed to each individual's human right to travel. When health professionals travel to receive training and then return to apply their skills, there are advantages to the home country. Additionally, emigrants of all social classes from poor countries typically send funds home to relatives, although sub-Saharan African remittances, at less than USD 5 billion, comprise the lowest dollar amounts of any other poor world region [8]. Further, it must be noted that individuals having benefit of public funds for their medical training are sending their remittances home to private parties with no direct gain for the health or education systems.

Immigration theory informs us that "push factors" prompt professionals to leave poor countries in favor of settling in higher income countries [22]. Negative factors in the sending countries include insufficient suitable employment, lower pay, unsatisfactory working conditions, poor infrastructure and technology, lower social status and recognition, and repressive governments. Simultaneously, "pull factors" in wealthier countries systematically attract physicians. These include training opportunities, higher living standards, better practice conditions and more sophisticated research conditions.

The "world systems framework theory" stresses the more permeable barriers between and among countries created by the standardized curriculum and English language used in world medical schools, the use of common research methods and shared scientific knowledge, the easy articulation of requirements of practice across countries, and the weakened nationalism that occurs as a result of professional training [23]. Other theories characterize migration as a decision of family units, rather than individuals, emphasizing the insurance nature of establishing what are, in effect, "branch offices" in multiple locations [24].

Given the enduring migration from poor countries to rich ones, only likely to increase with the international liberalization of trade in health services [25], concerns for global health require the maintenance of an adequate health workforce in poor countries.

## Methods

To describe the numbers and types of physicians practicing in the USA who earned medical degrees in Africa, we performed a cross-sectional study using the 2002 American Medical Association Physician Masterfile [26]. This data set contains detailed information on all 771 491 active physicians who were licensed to practice medicine in the year 2002 (excluding those physicians employed by federal entities such as the Veterans Administration, federal prisons or the military).

We reviewed these data for all physicians in the USA who received their training in sub-Saharan Africa (those 47 countries south of the Saharan desert on the African continent). These data included year of birth, gender, year of medical school graduation, name of medical school, current practice location, specialty of practice, and practice activity (office-based, hospital-based, in residency, or conducting teaching or research). Birth country information is missing for 68% of those who graduated from a sub-Saharan African medical school, so we did not analyze birth country data. To detect changes in migrant waves over time, we analyzed the data by cohorts, categorizing physicians who had graduated from medical school during four periods: before 1970, during the 1970s, during the 1980s, and 1990 and beyond.

We linked geographic data about practice locations to a four-category, rural-to-urban status and taxonomy, a condensed version of the Rural-Urban Commuting Area (RUCA) codes, to determine whether these physicians are practicing in rural or urban areas. RUCAs are a census tract-based classification scheme, that have also been adapted for zip codes, combining USA Census population data with work commuting information to characterize the types of rural and urban status [27].

Research colleagues in Canada and the UK provided some data on sub-Saharan African physicians in their countries, as well.

## Results

A total of 179 978 (23.3%) of the 771 491 active non-federal physicians in the USA in the year 2002 received their medical qualification in another country. The largest portion of these, or 115 835 physicians, originate from low and lower-middle income nations, as defined by the World Bank. Indeed, the most frequent countries of origin of IMGs in the USA include India (36 634), the Philippines (17 755), Mexico (10 404), and Pakistan (8563). Canadian physicians conventionally are not included in the IMG count because the body that accredits USA medical schools (the Liaison Committee on Medical Education (LCME)) offers reciprocal accreditation to Canadian medical schools (accredited by the Committee on Accreditation of Canadian Medical Schools). Canadians are, however, still subject to relevant immigration requirements.

Sub-Saharan African medical schools in 22 countries have trained approximately 5334 physicians currently practicing in the USA. Only nine nations, however, have lost more than 40 physicians each (see Table 2). Some 86% are from three countries (Nigeria, South Africa and Ghana). Nigeria, with more than twice the population of any other country in the region and 16 medical schools, has lost 2158 physicians who are now practicing in the USA; South Africa, with eight medical schools, has lost 1943 physicians; and Ghana, with three medical schools, has lost 478 physicians to the USA. By region, West Africa lost 2697 physicians and Southern Africa 1943. It is also suspected there are many more physicians from these countries working in the USA, although they are not licensed as physicians.

**Table 2:** Country of medical school of sub-Saharan African international medical graduates (IMGs) in the United States and Canada

**Country of training**	**Number of African-trained IMGs in USA^1^**	**Number of African-trained IMGs in Canada^2^**	**Number of physicians remaining in home country^3^**	**% of total African-trained now in USA or Canada^4^**
Nigeria	2158	123	22 894	9
South Africa	1943	1845	23 844	14
Ghana	478	37	1210	30
Ethiopia	257	9	1564	15
Uganda	133	42	722	20
Kenya	93	19	4001	3
Zimbabwe	75	26	1694	6
Zambia	67	7	676	10
Liberia	47	8	72	43
Other 12 countries*	83	35	12 912	1
Total/Average	5334	2151	69 589	10

1. American Medical Association: Physicians' professional record (AMA-PPD). 2002

2. Buske, Lynda. Associate director of research, Canadian Medical Association. Personal communication. February 3, 2003.

3. Number of physicians from the World Health Organization. Available at:

Calculation: [(Col. 1 + Col. 2)/ (Col. 1 + Col. 2 + Col. 3)]*100 = percent

* Other 12 countries with at least one graduate in the United States.

An analysis by school indicates only ten medical schools produced 79.4% of the sub-continent's graduates who are practicing in the USA. The medical schools most frequently attended by Sub-Saharan African IMGs in the USA include the University of the Witwatersrand (South Africa, 1053 physicians), the University of Cape Town (South Africa, 655), the University of Ibadan (Nigeria, 643), the University of Lagos (Nigeria, 429), the University of Nigeria (Nigeria, 394), the University of Ghana (Ghana, 389), Addis Ababa University (Ethiopia, 200), the University of Benin (Nigeria, 183), the University of Ife (Nigeria, 156), and the University of Pretoria (South Africa, 132), for a total of 4234 physicians.

An analysis of the numbers of sub-Saharan African IMGs coming to the USA in each of the last decades illustrates that it takes some time between graduation and emigration. The number of recent graduates currently in a USA residency program is higher than those in previous decades because of the obvious correlation between age and career stage.

Among sub-Saharan physicians in the USA, 78.3% are male. The picture is changing over time, however. Of the cohort who were trained in 1969 or earlier, 90% were male, but now only 66.3% of those who graduated from medical school in 1990 or later are male (see Table 3).

**Table 3:** Characteristics of sub-Saharan African international medical graduates in the United States by graduation year cohort

	**1969 or earlier**	**1970–1979**	**1980–1989**	**1990–2000**	**OVERALL**
Number	720	1167	2268	1179	5334
Currently in residency (%)	0	2.3	17.6	59.7	21.2
Gender (% male)	90.0	86.5	76.7	66.3	78.3
Generalists (%)	19.0	28.3	47.8	57.9	41.9
current practice location:					
Urban (%)	95.3	94.1	93.7	95.7	94.4
Large rural (%)	2.4	3.3	3.6	2.6	3.1
Small rural (%)	1.7	2.1	1.8	1.1	1.7
Isolated rural (%)	0.7	0.6	0.9	0.6	0.7

Note: includes residents.

Source of data: American Medical Association: Physicians' professional record (AMA-PPD) 2002.

The average age of sub-Saharan African physicians in the USA is 43 years, compared to 46 years for all USA physicians. Forty two percent of sub-Saharan African physicians in the USA are under 40 years, and another 32% are between 40 and 50. Among the large contributing countries, Nigerian physicians are the youngest cohort (63% are under 40), and South Africans are the oldest (only 20% are under 40).

A higher proportion of sub-Saharan physicians were in residency training programs (21.2%) than were USA physicians (14.1%), because many emigrate specifically for that reason. While 41.9% are in generalist specialty areas, compared to 34.7% of USA-trained physicians, the number has been rising with each new cohort. Table 3 illustrates that 57.9% of those trained in the 1990s selected a generalist practice specialty, compared to 28.3% of those trained in the 1970s. This apparently rising interest in generalist practice may be an artifact, however, as a prerequisite to internal medicine specialization is training in general internal medicine.

While 31.6% of all sub-Saharan African physicians in the USA are identified as family practitioners or general internists, it may be that this ratio of generalists will increase, as 45.4% of those in residency programs are in those two specialties. The next largest specialty groups are pediatrics (9.7%), psychiatry (5.5%), anesthesiology (5.4%), obstetrics and gynecology (3.3%) and general surgery (3.0%).

**Figure 1:**
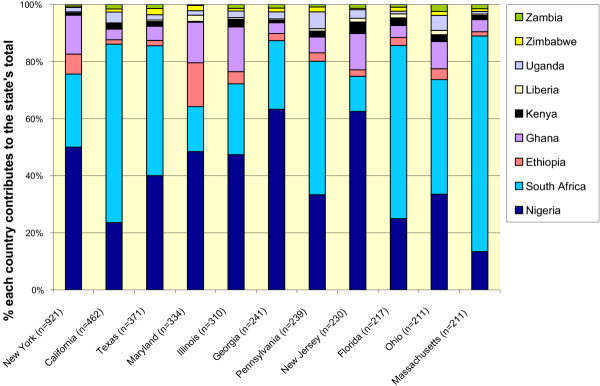
Origin and distribution of African-trained physicians in the 11 US states with the most such physicians

Urban areas attracted 93% of sub-Saharan African physicians (compared to 90.9% of other IMGs), even after excluding residents, who are typically based in urban teaching hospitals. Graduates of USA medical schools distribute themselves similarly, with 87% of USA-trained physicians in urban areas, even though a smaller 81% of the population lives in urban areas [27]. The states attracting the largest numbers of sub-Saharan Africa physicians include New York, California, Texas, Maryland, Illinois, Georgia, Pennsylvania, and New Jersey (see Figure 1). These are the same states that draw the largest portion of immigrant physicians generally.

The 1943 physicians trained in South African medical schools are somewhat different from their fellow African trainees from the subcontinent. They are older, more of them are male, they are typically white (94%) and they are more often in a subspecialty practice. This may reflect a particular wave of physicians seeking subspecialty training and practice opportunities in the USA during the political turmoil South Africa experienced in the 1970s and 1980s. It is unlikely they are seeking training abroad that is unavailable in their home country, as the medical training opportunities in South Africa are quite comprehensive.

## Discussion

The 5000-plus physicians trained in sub-Saharan Africa who have migrated to the USA comprise only a small proportion of the total number of IMGs practicing in the USA. However, their relatively small numeric addition to the USA medical workforce contrasts markedly with the impact of their migration on the medical workforce in sub-Saharan Africa. Moreover, in absolute terms, the USA has drawn more of the medical workforce of Africa than either Canada, with 2151 African graduates (Lynda Buske, Canadian Medical Association, personal communication, 2/3/03), or the UK, with 3451 (Bonnie Sibbald, University of Manchester, personal communication, 1/24/03), largely because of the relative size of the health care system in the USA.

Including the USA, the UK, and Canada, then, 10 936 physicians trained in sub-Saharan Africa are practicing in the three countries, a number that represents 12% of all African physicians. We expect there are many more African physicians who are in the UK, as well, as our figures from there include only those who arrived post-1992. While some of the physicians in residency training will return home, there are unknown others who could be practicing medicine at home but were not able to get licenses abroad and therefore are engaged in other occupations. Almost all the medical schools sending graduates to the USA provide their instruction in English.

Our study provides some measures of sub-Saharan Africa physician migration to the USA. A next step is to collect data from other developed countries and begin to create a physician migration data set from multiple countries. Migration from country to country within the subcontinent is also worthy of further examination, and it would be useful to identify the relationship between country of birth and country of training. There are likely to be some medical schools that draw students from several African countries. It should be noted, as well, that there are physicians practicing in Africa who did not train there, most prominently Cubans.

Some sub-Saharan countries lose a larger proportion of their physicians to the USA than others. For example, while Ghana has a reported 1210 practicing physicians in its country, 478 graduates of Ghanaian medical schools are practicing in the USA. Without even considering those who have migrated to other countries, these 478 Ghanaian graduates in the USA represent 30% of Ghana's potential medical workforce (see Table 2). If none of those had come to the USA, the physician-to-population ratio in Ghana would rise from 6.2 to 8.7 per 100 000, or a 40% increase. By comparison, South Africa has lost 14% of its potential workforce to the USA and Canada.

The migration of physicians from sub-Saharan Africa represents a lost investment of significant training costs, since graduates of medical schools in Africa are likely to have contributed financially to only a small portion of the costs of their medical education [28]. Medical education is estimated to cost Ghana about USD 9 million per year and Nigeria USD 20 million (Hagopian & Ofosu, et al. *The flight of physicians from West Africa: views of African physicians and implications for policy*, unpublished 2003).

The United Nations Commission for Trade and Development has estimated that each professional leaving Africa costs the continent USD 184 000, or USD 4 billion a year – one third of official development funds to Africa [29]. The loss of trained health personnel also contributes to a general decline in average incomes, as physicians generate skilled health system jobs beyond their own. Lost tax revenues from absent physicians represent significant losses as well.

Ostensibly, the USA welcomes IMGs for two purposes. First, as a form of foreign aid, it provides specialty training that physicians can take back to their home countries for the benefit of residents of those nations. Second, IMGs fill positions in specialties and locations that are less attractive to their USA counterparts, and may help to correct physician maldistribution in some rural or underserved areas of the USA. (There are several federal agencies, along with state health departments who "sponsor" physicians who have completed their residency training in the USA on J-1 exchange – or "student"- visas. These sponsorships allow foreign national physicians to gain approval from the State Department and the USA Citizenship and Immigration Services to waive J-1 visa requirements that would otherwise require them to return to home countries for at least two years. In exchange for this waiver, physicians find employment with a health agency or private physician in a health professional shortage area.)

Longitudinal tracking of physicians entering the USA has indicated, however, that few IMGs ever leave the USA after arriving for residency training [30], and there is conflicting evidence about whether IMGs are more likely to practice in safety net practices for low-income and underinsured people [21,31,32].

Attracting physicians to rural practice in most countries is difficult, and is accomplished only through a careful set of policies designed to provide incentives for rural service. In most poor as well as rich countries, physicians are concentrated around urban hospitals that offer tertiary care, even though more rational service delivery systems might focus on a geographically decentralized system of primary and preventive care. Poor countries that offer medical training to produce too many physicians with highly technical skills, some of whom who cannot find satisfying jobs, may further contribute to physician migration [33]. India and the Philippines, for example, clearly over-produce physicians who are intended for an international market.

Our findings show African physicians are unlikely to select small or remote rural practice opportunities in either their home countries or in the USA, but the preponderance of African physicians in American inner-city underserved areas may to some extent be helpful to USA needs by boosting the number of minority physicians in the urban health workforce. The growing number of African immigrant female physicians follows the trend for increasing numbers of female physicians trained in the USA, and probably has similar implications. Some researchers have found, for instance, that female physicians are less likely to practice in rural areas [34].

While the sub-Saharan Africa region as a whole loses many of its physicians, it is apparent that a small handful of medical schools is the sources of the majority of this migration. Ten medical schools in four countries – South Africa, Nigeria, Ghana and Ethiopia – produce 79.4% of the émigré physicians to the USA, out of a total of 87 medical schools in the region. This suggests policy approaches to reducing the "brain drain" from Africa could be targeted at only these few countries or medical schools, a less daunting task than addressing the problem in 47 different countries.

Medical migration results from the complex interaction of myriad social, legal and economic forces. Single country policies are unlikely to alter the flows significantly. Even if the USA acknowledges that that it benefits from luring medical professionals here for whose medical school training we do not pay, solutions that would be compatible with social justice principles are not clear. Furthermore, if all African doctors returned to their home countries today, they would not necessarily find satisfactory employment opportunities in cash-strapped health systems.

## Conclusions

The 57th World Health Assembly, in 2004, adopted a resolution to urge member countries to develop strategies to mitigate the adverse effects of migration of health workers; to develop policies that could provide incentive for health workers to remain in their countries; and, among other issues, requests WHO to help countries set up information systems to monitor the movement of health resources for health, and to include human resources for health development as a top-priority program at WHO from 2006 to 2015 [35].

In an ideal world, freedom of movement is a universal right for individuals, as there is ostensibly no rational reason why anyone would have a stronger right to be in any place more than anyone else [36]. Today, however, differences in wealth between countries create flows of educated people seeking better opportunities far from home. One result is that resource-strapped African (and other poor) countries have invested significant resources in educating health professionals who will never serve the populations that were taxed (or took out high-interest loans from international lenders) to pay for their training.

Importing health professionals from poor countries to provide care in rich countries is not consistent with a rational workforce policy rooted in social justice principles. In the short run, Mullan [37] and others are right to recommend that the USA expand its incentives to USA graduates to practice in rural and underserved areas through the National Health Service Corps and other programs. Grumbach [18] recommends reducing the number of excess residency training positions by limiting the Medicare subsidy.

In response to a recent report by the USA Physicians for Human Rights [38], *The New York Times *editorialized that the "obvious long-term solution to the medical brain drain is for wealthier countries to reimburse Africa's health and educational systems for the cost of poaching their professionals, and to greatly increase the financing and technical help for Africa's health systems" [39]. This unprecedented attention to the issue of the African medical brain drain in a major USA publication, coupled with a radical call for reparations, suggests USA policy makers may be called to address this issue.

The same Physicians for Human Rights report that prompted *The New York Times *response made a strong recommendation that the International Monetary Fund, World Bank, and other donors refrain from withholding loans or grants from countries that increase their spending on "health, education and other sectors and activities needed to promote human development, including to enhance salaries to health staff or to hire new health personnel."

One of the major limitations African nations face in addressing their health workforce problems is the lack of reliable data on how many health workers have graduated from their schools, how many are working in the country and in what locations, and how many have emigrated. It is urgent that poor countries put together the information systems required to track these data, as a basis for workforce policy and investment decisions.

And, finally, the fact that so few medical schools generate the vast majority of emigrants creates an opportunity to focus attention in a strategic way. These schools might be enticed to redirect their missions towards producing graduates who intend to serve their own countries. This would likely require curriculum changes, admissions policy changes, and a change in faculty culture to ensure that emigration is not promoted as a mark of prestige.

## Competing interests

The author(s) declare that they have no competing interests.

## Authors' contributions

AH conceived the project, designed the research and wrote the paper. MJT and KEJ actively participated in the conceptualization and re-writing of the paper. MF conducted the data management and analysis. LGH provided guidance, advice and editing assistance. All authors read and approved the final manuscript.
